# Supplementary material for: Structural Characterization of Calcium-Dependent Calmodulin-Calmidazolium Binding Using Capillary Vibrating Sharp-Edge Spray Ionization-Based Native Mass Spectrometry and In-Droplet Hydrogen–Deuterium Exchange Mass Spectrometry
Source: ACS Omega. 2026 Aug 31;11(36):54578–92. doi: 10.1021/acsomega.6c07079 (PMC13584159; doi:10.1021/acsomega.6c07079)
Supplement: Supplementary file 1 [file ao6c07079_si_001.pdf]

Structural Characterization of Calcium-Dependent Calmodulin-Calmidazolium Binding using Capillary Vibrating Sharp-Edge Spray-based Native Mass Spectrometry and In-Droplet Hydrogen Deuterium Exchange Mass Spectrometry.

Sohag Ahmed[1*], Axel Woehrling[2], Peng Li[3], Stephen J. Valentine[1,4], and Kevin C. Courtney[2,5,*]

[1]Department of Chemistry, West Virginia University, Morgantown, WV 26506, USA
[2]Department of Biochemistry and Molecular Medicine, West Virginia University, Morgantown, WV 26506, USA
[3]Department of Chemistry, Lehigh University, 6 East Packer Avenue, Bethlehem, PA 18015
[4]Department of Arts and Science, University of Louisville, Louisville, KY, 40292, USA
[5]Department of Molecular and Cellular Biology, University of Guelph, Guelph, ON N1G 2W1, Canada
*Corresponding authors: sa00123@mix.wvu.edu; kevin.courtney@uoguelph.ca

Supporting Information

Supplementary Method: cVSSI Device Fabrication and Troubleshooting Guide for cVSSI-nMS and in-droplet HDX-MS 2-3

Figure S1 4

Figure S2 5

Figure S3 6-7

Figure S4 8-9

Figure S5 10-11

Figure S6 12

Table S1 13-14

Table S2 15

Table S3 16

Table S4 17

Table S5 18

Table S6 19

Supplementary Method

cVSSI Device Fabrication.

Each device was constructed by affixing a piezoelectric transducer (7BB-27-4L0, diameter = 27 mm, Murata) on one end of a glass slide coverslip (width × length = 25 × 60 mm) (VWR, Radnor, PA) with epoxy glue (Devcon). The emitter tip was made of a glass capillary (4 mm × 1 mm), which was pulled using a P2000 micropipette puller (Sutter Instrument, Novato, CA) to obtain tips of 10 to 20 μm ID. The opening orifice size of the pulled emitter tips was measured using a microscope. This emitter tip was attached with glue to the distal edge of the glass slide at 60° angle as described previously. Thin-wall poly(tetrafluoroethylene) (PTFE) tubing, about 40 centimeters in length, was slip fit onto the blunt end of the attached pulled-tip capillary. A tiny puncture was made close to the end of the PTFE tubing using a BD Precision Glide needle (0.3 mm × 13 mm), through which a short Pt wire of approximately 5 cm long and 20 μm in diameter (Alfa Aesar, Ward Hill, MA) was carefully inserted. To secure the Pt wire and prevent any leakage, a small quantity of epoxy glue was applied to the outer surface of the tube. The Pt wire serves to supply DC voltage to the solution being infused. The opposite end of the tubing was affixed to the needle (BD 0.3 mm × 13 mm) luer lock using epoxy glue. The luer lock was then firmly attached to the syringe (BD 1 mL), enabling direct infusion of samples facilitated by a syringe pump.[1,2]

Troubleshooting Guide for the cVSSI-nMS and in-droplet HDX-MS

Issue Observed	Possible Cause	Practical Adjustment
No spray formation	1. Emitter tip and PTFE tube clogging.<br>2. Frequency and amplitude are not optimized.	1. After use, the device and PTFE tubing need to be cleaned using methanol and 0.1% (v/v) formic acid for at least 10 minutes by just spraying with the syringe pump.<br>2. Frequency and amplitude should be optimized by spraying water before introducing the analytes.
Low signal intensity	1. Poor emitter-to-inlet alignment<br>2. Weak plume formation<br>3. Partial clogging of the emitter<br>4. Inconsistent flow rate	1. Adjust the emitter tip position relative to the MS-inlet.<br>2. Optimize the frequency and amplitude<br>3. Inspect the emitter tip for clogging or damage<br>4. Maintain stable flow from the syringe pump.
Strong salt/adduct formation	1. Excess non-volatile salts<br>2. Incomplete buffer exchange	1. MS-friendly buffers such as ammonium acetate (50-100 mM).<br>2. Buffer exchange of the protein before analysis.
Difficulty assigning metal-bound states	Multiple Ca^{2+} adducts; Non-specific metal association	Confirm complete Ca^{2+} saturation of the protein using different concentrations (low to high) of calcium acetate against a fixed concentration of protein.
Low ligand binding signal	Insufficient ligand concentration; incomplete Ca^{2+} loading; ligand precipitation; ligand loss during ionization; poor mixing	Confirm Ca^{2+} saturation conditions, prepare fresh ligand stock, maintain consistent final DMSO concentration (< 2% of the total analyte volume)
Inconsistency in HDX-MS	Variable plume overlap, emitter position, flow rate, spray stability, or data-processing boundaries	Fix emitter positions (Perpendicular to each other); use the same flow rates and acquisition settings, collect replicate spectra under identical conditions, and apply consistent peak-integration or centroiding criteria
Difficulty comparing HDX between bound and unbound species	Bound and unbound peaks overlap; poor signal-to-noise	Compare bound and unbound species within the same charge state, exclude ambiguous or overlapping peaks, and report only reproducible charge states with clear isotopic envelopes

For reproducible measurements, the spray plume, emitter position, flow rate, and MS source conditions were optimized before each experiment, and undeuterated controls were acquired under the same instrumental conditions used for in-droplet HDX-MS.

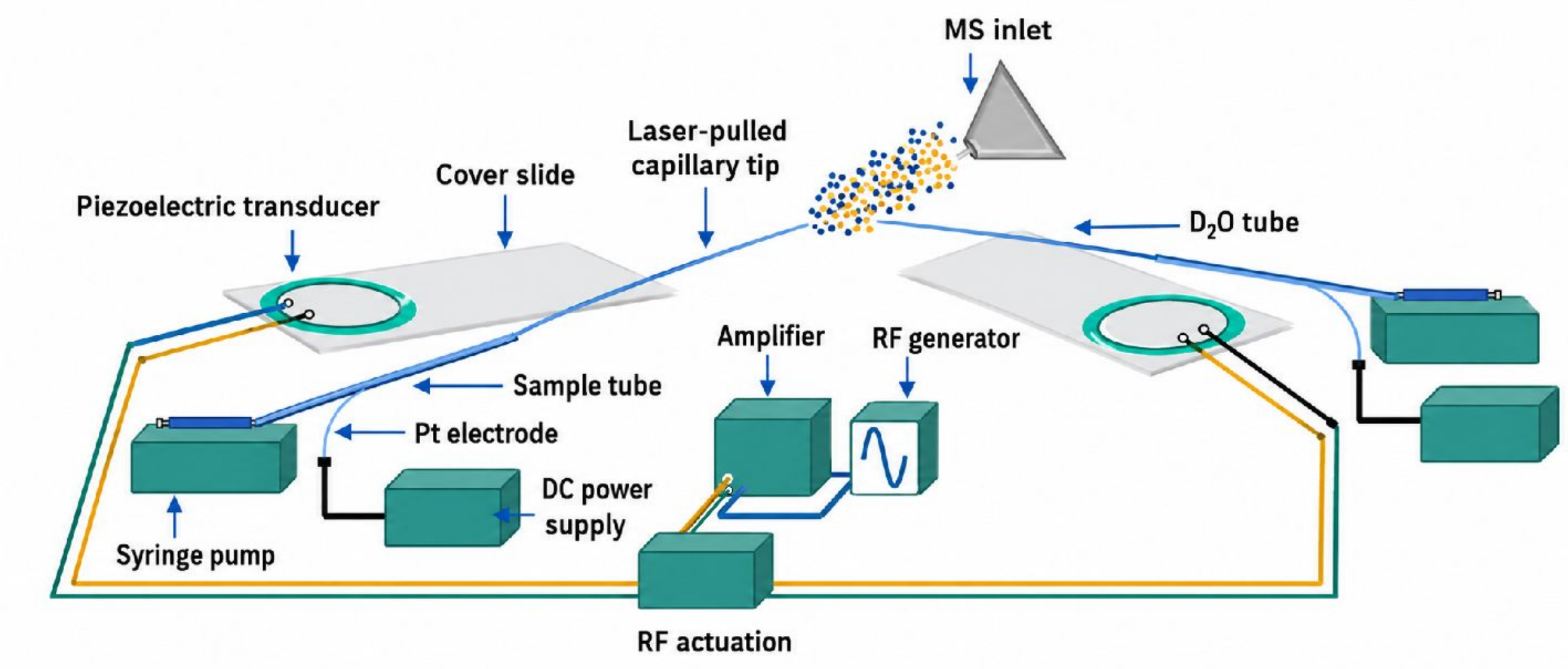

Figure S1. Dual emitter setup containing two cVSSI devices for in-droplet HDX-MS experiments. The left device produces droplets of the analyte solution (blue dots) and the right device produces droplets D_2O (orange dots). Droplet mixing occurs in flight before entering the MS-inlet, enabling rapid hydrogen-deuterium exchange. Solutions are delivered to each device using syringe pumps, and droplets are produced from vibrating laser-pulled capillary tips driven by piezoelectric transducers. An RF generator and amplifier provide the actuation signal required for capillary vibration, while a DC power supply applies voltage to the analyte solution through a platinum wire (Pt electrode) to facilitate ionization.[3,4]

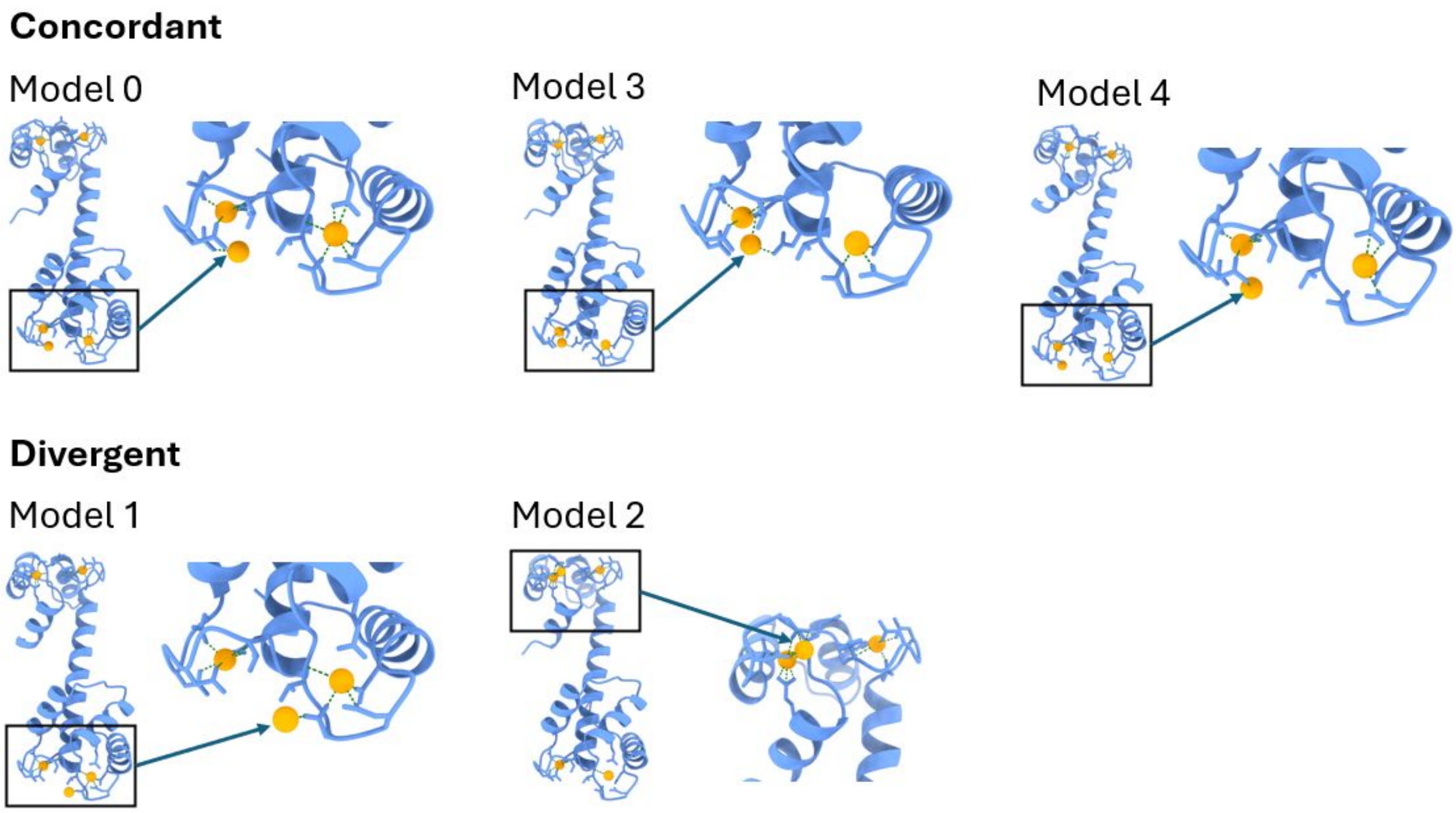

Figure S2. Five AI-predicted CaM 5+ Ca^{2+} structural models. CaM is represented by a cornflower blue ribbon, the Ca^{2+} ions are represented by orange spheres and the predicted interactions are represented by dashed green lines. Black rectangles show a zoom of the region where the fifth Ca^{2+} ion binds, and green arrows point towards the fifth Ca^{2+} ion. Models 0, 3, and 4 display a similar binding site for the fifth Ca^{2+} ion and are regrouped as “concordant”. Model 1 displays a binding site close to the one of the models 0, 3, 4 but that differs from those concordant models. Model 2 displays a completely different binding location compared to all the other models. Model 1 and model 2 are then regrouped as “divergent”.

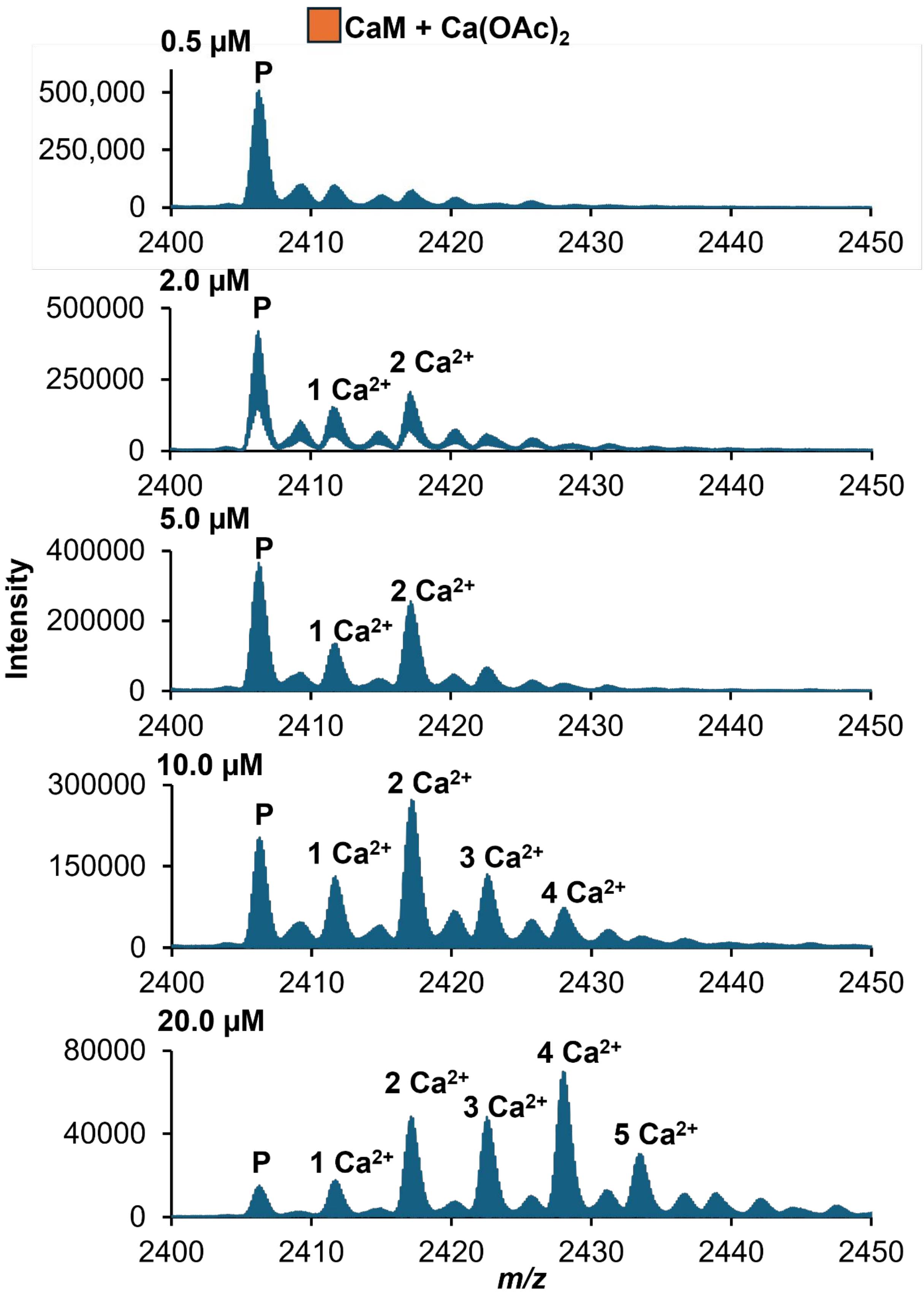
CaM + Ca(OAc)2
0.5 μM
P
500,000
250,000
0
2400
2410
2420
2430
2440
2450
2.0 μM
P
2 Ca2+
1 Ca2+
500000
250000
0
5.0 μM
P
2 Ca2+
1 Ca2+
400000
200000
0
Intensity
10.0 μM
P
2 Ca2+
1 Ca2+
3 Ca2+
4 Ca2+
300000
150000
0
20.0 μM
P
1 Ca2+
2 Ca2+
3 Ca2+
4 Ca2+
5 Ca2+
80000
40000
0
m/z

Figure S3. **Zoomed-in native mass spectra of 7+ charge state of Calmodulin at increasing concentrations of $Ca(OAc)_2$**. The CaM concentration was maintained at 0.5 μM, while the $Ca(OAc)_2$ was titrated at CaM: $Ca(OAc)_2$ ratios of 1:1, 1:4, 1:10, 1:20 and 1:40. Increase concentration of $Ca(OAc)_2$ resulted in progressive enrichment of Ca^{2+}-bound species. All four calcium-binding sites of the CaM protein were occupied at a ratio of CaM (0.5 μM):$Ca(OAc)_2$ (20 μM) = 1:40. P and Ca^{2+} peaks represent unbound and Ca^{2+}-bound CaM species, respectively.

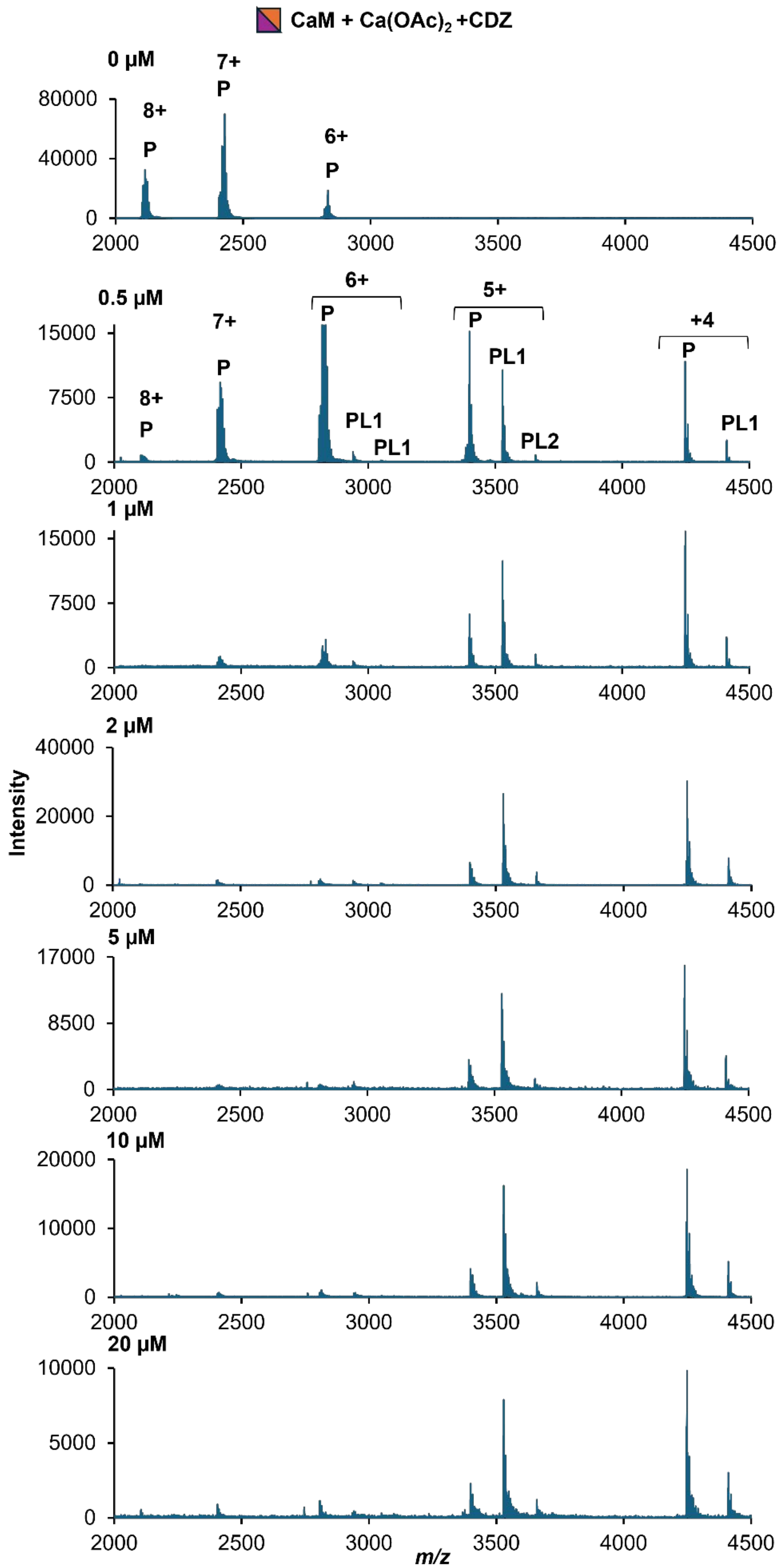

CaM + Ca(OAc)$_2$ +CDZ
0 µM
8+
P
7+
P
6+
P
0.5 µM
8+
P
7+
P
6+
P
PL1
PL1
5+
P
PL1
PL2
+4
P
PL1
1 µM
2 µM
5 µM
10 µM
20 µM
Intensity
m/z

Figure S4. Native mass spectra of CaM with increasing CDZ concentrations. Spectra are shown from top to bottom with increasing CDZ concentration. The CaM and $Ca(OAc)_2$ concentrations were maintained at 0.5 μM and 20 μM, respectively, throughout the experiment. Peaks correspond to the observed charge states and associated protein species at each condition. P, PL1, and PL2 correspond to populations of CaM that are unbound, one and two CDZ molecules bound, respectively.

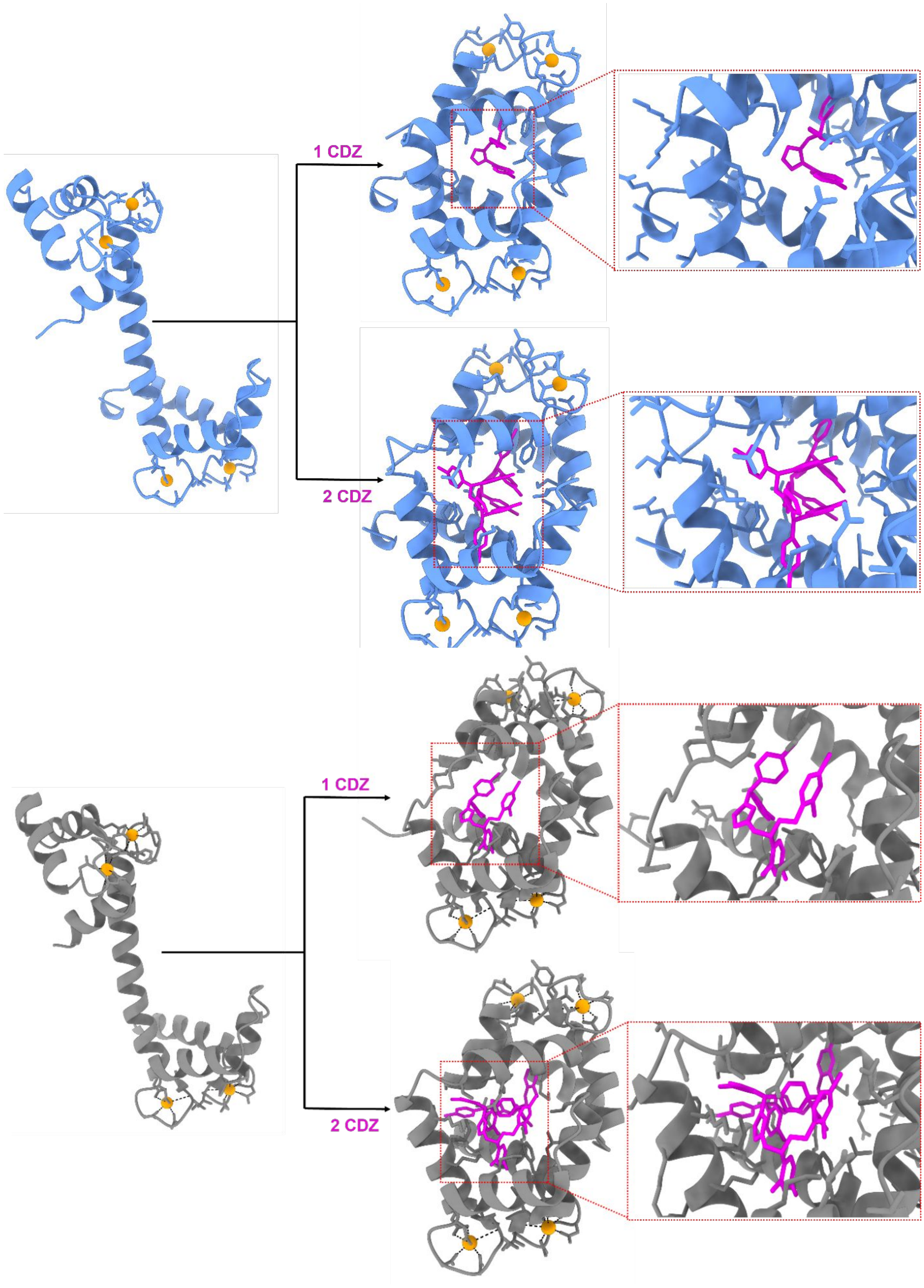

Figure S5. Comparison between predicted and experimental crystal structures of

Ca^{2+}-bound CaM complexed with one and two CDZ molecules. The top panel shows Chai-1 predicted structures, and the bottom panel shows structures obtained from the Protein Data Bank (PDB). In both cases, the left panel shows Ca^{2+}-bound, the middle panel shows one and two CDZ-bound CaM. The right panel is the enlarged view of the CDZ binding region. The CaM is shown in ribbon representation, Ca^{2+} ions are represented as orange spheres, and CDZ in magenta sticks. The structures obtained from the Protein Data Bank are Ca^{2+}-bound CaM (PDB ID: 1CLL[5]), one CDZ-bound CaM (PDB ID: 7PSZ[6]), and two CDZ-bound CaM (PDB ID: 7PU9[6]).

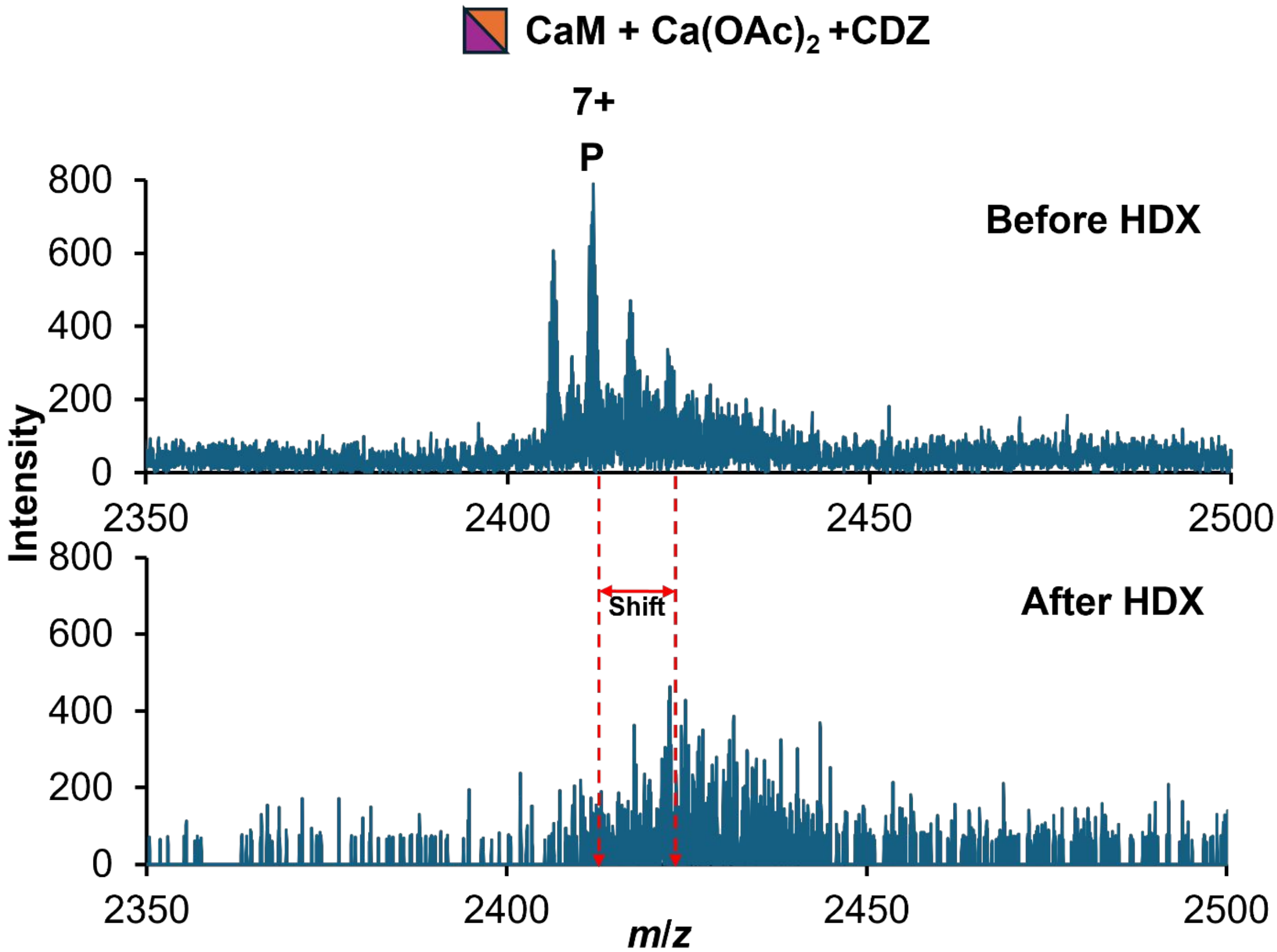

Figure S6. Zoomed-in mass spectra of 7+ CaM species before and after HDX. The red dashed lines indicate the shift in *m*/*z* of CaM species after HDX, resulting in deuterium incorporation. P corresponds to unbound protein peaks.

Table S1. Comparison between Chai-1 predicted structures and experimental crystal structures. The Chai Discovery[7] generated structural models were compared to their corresponding experimental crystal structures using the MatchMaker tool in UCSF ChimeraX[8] (bb chain pairing, Needleman–Wunsch alignment algorithm, BLOSUM-62 similarity matrix, 0.3 secondary structure fraction, 18/18/6 gap open (HH/SS/other), 1 gap extend, ((6-9-6), (6-6), (4)) (HSOxHSO) secondary structure matrix, and 2 Å iteration cutoff). Chai Discovery generated five models (Model 0-4) for each of the following structures: CaM + 4 Ca^{2+}, CaM + 1 CDZ + 4 Ca^{2+}, and CaM + 2 CDZ + 4 Ca^{2+}. The predicted models were compared with their corresponding experimental crystal structures from the Protein Data Bank: CaM + 4 Ca^{2+} (PDB ID: 1CLL[5]), CaM + 1 CDZ + 4 Ca^{2+} (PDB ID: 7PSZ[6]), and CaM + 2 CDZ + 4 Ca^{2+} (PDB ID: 7PU9[6]). The pruned RMSD (Å) is the root-mean-square deviation of CaM atoms positions after removal by iterative exclusion of residue pairs that are too far apart (iteration cutoff: 2 Å). The lower the pruned RMSD value, the better the structural fit of the best-aligned core. The number of pruned atom pairs corresponds to the number of atom pairs that were kept during the iterative exclusion step of the RMSD calculation. The coverage (%) highlights the fraction of CaM residues kept after pruning and is calculated as follows: Coverage=[pruned pairs / total pairs] × 100. The higher the coverage, the more structurally complete the alignment. The PDB CaM crystal structures had different residue numbers, which we considered for the calculations (CaM + 4 Ca^{2+} / PDB ID 1CLL had 144 pairs, CaM + 1 CDZ + 4 Ca^{2+} / PDB ID 7PSZ had 145 pairs, and CaM + 2 CDZ + 4 Ca^{2+} / PDB ID 7PU9 had 144 pairs). For each structure, the model with the lowest pruned RMSD and the model with the highest coverage are highlighted to characterize structural fitness and completeness.

Structure	Model	Pruned RMSD (Å)	Number of pruned atom pairs	Coverage (%)
CaM + 4 Ca^{2+}	Model 0	1.205	111	77.1
	Model 1	**0.865**	81	56.3
	Model 2	1.031	130	**90.3**
	Model 3	1.188	88	61.1
	Model 4	1.155	113	78.5
CaM + 1 CDZ + 4 Ca^{2+}	Model 0	1.215	78	53.8
	Model 1	1.251	84	57.9
	Model 2	1.159	80	55.2
	Model 3	**0.606**	54	37.2

	Model 4	1.263	92	**63.4**
CaM + 2 CDZ + 4 Ca^{2+}	Model 0	**1.151**	119	**82.6**
	Model 1	1.192	105	72.9
	Model 2	1.178	108	75.0
	Model 3	1.168	106	73.6
	Model 4	1.193	109	75.7

Table S2. Fraction of CaM bound calculated using equation (1) from the main article for two resolved and reproducible (+5 and +4) charge states across increasing CDZ concentrations. Apparent K_d was determined by fitting the experimental data to the Langmuir binding model.[9] The sum of squared residuals (SSR) assesses the agreement of the fit between experimental and predicted values.

Ligand concentration, L (μM)	Fraction bound	Predicted Y	Residual^2	Fitting Parameters
0	0	0	0	B_{max}
0.5	0.342738829	0.360658711	0.000321122	0.526
1	0.44327044	0.427913197	0.000235845	
2	0.488552227	0.471913676	0.000276841	
5	0.503708699	0.502942956	5.86361E-07	
10	0.509495037	0.514213142	2.22605E-05	
20	0.526183644	0.520039791	3.77469E-05	K_d (μM)
		SSR	0.000894402	0.229221

Table S3. Fraction of CaM bound calculated using equation (1) from the main article for two resolved and reproducible (+5 and +4) charge states across increasing CDZ concentrations. Apparent Kd was obtained by fitting the binding data to the Quadratic binding model.[10] The sum of squared residuals (SSR) assesses the agreement of the fit between experimental and predicted values.

**Ligand concentration, L_0 (μM)**	**Fraction bound**	Predicted Y (Quadratic)	Residual	Residual^2	Fitting Parameters
0	0	0	0	0	Protein Conc.(P_o)
0.5	0.342739	0.334043	0.008695786	7.56167E-05	
1	0.443270	0.446378	-0.003107523	9.6567E-06	0.5 μM
2	0.488552	0.494985	-0.006432812	4.13811E-05	B_{max}
5	0.503709	0.51756	-0.01385103	0.000191851	0.53
10	0.509495	0.524022	-0.014526619	0.000211023	
20	0.526184	0.527069	-0.000885567	7.84229E-07	K_d (μM)
			SSR	0.000530312	0.108446

Table S4. Apparent K_d values obtained from triplicate CaM-CDZ native MS titration experiments using Langmuir and quadratic binding models.

Experiment	Langmuir K_d (nM)	Quadratic K_d (nM)
Replicate 1	284.52	142.54
Replicate 2	229.22	108.45
Replicate 3	269.67	125.72
**Mean ± SD**	**261.14 ± 28.62**	**125.57 ± 17.05**

Table S5. Deuterium incorporation calculated from HDX envelope center (*m/z)* shifts for unbound and CDZ-bound CaM at the 7+ to 4+ charge states across triplicate experiments. The number of incorporated deuterium atoms was calculated for two binding sites observed in the 5+ species, where the differences in the number of incorporated deuterium were calculated relative to 1st CDZ-bound population and the percentage was calculated comparing unbound CaM population. For the 7+ charge state, the number of incorporated deuterium was calculated for the unbound protein population only.

**Experiments**	**Condition**	**After HDX m/z**	**Before HDX *m/z***	**Δ *m/z***	**Charge states**	**Incorporated deuteriums (D)**	**Reduction (D)**	**Reduction (%)**
1	Unbound	4256.78	4248.2	8.37	4	33.46	4.12	12.31
	Bound	4418.65	4411.31	7.34		29.34		
2	Unbound	4255.51	4248.2	7.33		29.32	3.02	10.29
	Bound	4417.89	4411.31	6.58		26.31		
3	Unbound	4255.91	4248.2	7.49		29.97	3.03	10.11
	Bound	4418.04	4411.31	6.73		26.94		
1	Unbound	3405.15	3398.92	6.23	5	31.16	7.81	25.07
	Bound	3533.87	3529.18	4.68		23.35		
2	Unbound	3405.37	3398.92	6.45		32.25	6.51	20.2
	Bound	3534.35	3529.18	5.16		25.74		
3	Unbound	3404.95	3398.92	6.02		30.12	6.24	20.73
	Bound	3533.97	3529.18	4.79		23.88		
1	Bound	3663.51	3659.48	4.03	5	20.14	3.21	10.30
2	Bound	3663.98	3659.48	4.49		22.47	3.27	10.14
3	Bound	3663.61	3659.48	4.12		20.61	3.27	10.86
1	Unbound	2835.56	2825.78	9.79	6	58.71	23.46	39.96
	Bound	2956.04	2950.16	5.87		35.25		
2	Unbound	2836.06	2825.78	10.28		61.71	22.07	35.76
	Bound	2956.77	2950.16	6.61		39.64		
3	Unbound	2835.77	2825.78	9.99		59.96	21.58	36.00
	Bound	2956.56	2950.16	6.39		38.37		
1	Unbound	2428.91	2418.84	10.07	7	70.51	No drug-bound species.	
2	Unbound	2428.50	2418.84	9.66		67.64		
3	Unbound	2428.46	2418.84	9.62		67.37		

Table S6. Quantitative summary of deuterium uptake for unbound and CDZ-bound CaM at the 6+, 5+, and 4+ charge states, with unbound 7+ CaM population included for comparison. Values were calculated from HDX envelope center m/z shifts across triplicate experiments. The difference and reduction percentages were calculated relative to the corresponding unbound CaM value for each replicate, except for the 2nd CDZ-bound population. For the 2nd bound population, the reduction percentage was calculated relative to the unbound and the difference was calculated relative to the 1st CDZ-bound population.

Charge state		Experiment	Deuterium uptake before CDZ binding (D)	Deuterium uptake after CDZ binding (D)	Difference (D)	Reduction (%)
4+		1	33.46	29.34	4.12	12.31
		2	29.32	26.31	3.02	10.29
		3	29.97	26.94	3.03	10.11
		**Mean ± SD**	**30.92 ± 2.22**	**27.53 ± 1.60**	**3.39 ± 0.63**	**10.90 ± 1.22**
5+	1st	1	31.16	23.35	7.81	25.07
		2	32.25	25.74	6.51	20.20
		3	30.12	23.88	6.24	20.73
		**Mean ± SD**	**31.18 ± 1.06**	**24.32 ± 1.26**	**6.86 ± 0.84**	**22.00 ± 2.67**
	2nd	1	23.35	20.14	3.21	10.30
		2	25.74	22.47	3.27	10.14
		3	23.88	20.61	3.27	10.86
		**Mean ± SD**	**24.32 ± 1.26**	**21.07 ± 1.23**	**3.25 ± 0.03**	**10.43 ± 0.38**
6+		1	58.71	35.25	23.46	39.96
		2	61.70	39.63	22.07	35.76
		3	59.95	38.37	21.59	36.00
		**Mean ± SD**	**60.12 ± 1.51**	**37.75 ± 2.26**	**22.37 ± 0.97**	**37.24 ± 2.35**
7+		1	70.51	**Not Applicable**		
		2	67.63			
		3	67.37			
		**Mean ± SD**	**68.51 ± 1.73**			

REFERENCES

(1) Ranganathan, N.; Li, C.; Suder, T.; Karanji, A. K.; Li, X.; He, Z.; Valentine, S. J.; Li, P. Capillary Vibrating Sharp-Edge Spray Ionization (cVSSI) for Voltage-Free Liquid Chromatography-Mass Spectrometry. *J. Am. Soc. Mass Spectrom.* **2019**, *30* (5), 824–831. https://doi.org/10.1007/s13361-019-02147-0.

(2) Sharif, D.; Foroushani, S. H.; Attanayake, K.; Dewasurendra, V. K.; DeBastiani, A.; DeVor, A.; Johnson, M. B.; Li, P.; Valentine, S. J. Capillary Vibrating Sharp-Edge Spray Ionization

Augments Field-Free Ionization Techniques to Promote Conformer Preservation in the Gas-Phase for Intractable Biomolecular Ions. *J Phys Chem B* **2022**, *126* (44), 8970–8984. https://doi.org/10.1021/acs.jpcb.2c04960.
(3) Sharif, D.; Rahman, M.; Mahmud, S.; Sultana, M. N.; Attanayake, K.; DeBastiani, A.; Foroushani, S. H.; Li, P.; Valentine, S. J. In-Droplet Hydrogen–Deuterium Exchange to Examine Protein/Peptide Solution Conformer Heterogeneity. *Rapid Communications in Mass Spectrometry* **2023**, *37* (16), e9593. https://doi.org/10.1002/rcm.9593.
(4) DeBastiani, A.; Majuta, S. N.; Sharif, D.; Attanayake, K.; Li, C.; Li, P.; Valentine, S. J. Characterizing Multidevice Capillary Vibrating Sharp-Edge Spray Ionization for *In-Droplet* Hydrogen/Deuterium Exchange to Enhance Compound Identification. *ACS Omega* **2021**, *6* (28), 18370–18382. https://doi.org/10.1021/acsomega.1c02362.
(5) Chattopadhyaya, R.; Meador, W. E.; Means, A. R.; Quiocho, F. A. Calmodulin Structure Refined at 1.7 Å Resolution. *Journal of Molecular Biology* **1992**, *228* (4), 1177–1192. https://doi.org/10.1016/0022-2836(92)90324-D.
(6) Léger, C.; Pitard, I.; Sadi, M.; Carvalho, N.; Brier, S.; Mechaly, A.; Raoux-Barbot, D.; Davi, M.; Hoos, S.; Weber, P.; Vachette, P.; Durand, D.; Haouz, A.; Guijarro, J. I.; Ladant, D.; Chenal, A. Dynamics and Structural Changes of Calmodulin upon Interaction with the Antagonist Calmidazolium. *BMC Biol* **2022**, *20* (1), 176. https://doi.org/10.1186/s12915-022-01381-5.
(7) Chai Discovery; Boitreaud, J.; Dent, J.; McPartlon, M.; Meier, J.; Reis, V.; Rogozhnikov, A.; Wu, K. Chai-1: Decoding the Molecular Interactions of Life. October 11, 2024. https://doi.org/10.1101/2024.10.10.615955.
(8) Goddard, T. D.; Huang, C. C.; Meng, E. C.; Pettersen, E. F.; Couch, G. S.; Morris, J. H.; Ferrin, T. E. UCSF ChimeraX: Meeting Modern Challenges in Visualization and Analysis. *Protein Science* **2018**, *27* (1), 14–25. https://doi.org/10.1002/pro.3235.
(9) Li, J.; Ma, X.; Wang, X.; Hu, X.; Fang, S.; Jin, G.; Liu, K.; Dong, Z. Mutations Found in Cancer Patients Compromise DNA Binding of the Winged Helix Protein STK19. *Sci Rep* **2024**, *14* (1), 14098. https://doi.org/10.1038/s41598-024-64840-9.
(10) Liu, W. H.; Zheng, J.; Feldman, J. L.; Klein, M. A.; Kuznetsov, V. I.; Peterson, C. L.; Griffin, P. R.; Denu, J. M. Multivalent Interactions Drive Nucleosome Binding and Efficient Chromatin Deacetylation by SIRT6. *Nat Commun* **2020**, *11* (1), 5244. https://doi.org/10.1038/s41467-020-19018-y.
